# *Bacillus subtilis* AC7 Fermentation on Rice Husk Substrate: A Sustainable Approach for Lipopeptide Biosurfactant Production

**DOI:** 10.3390/microorganisms14061288

**Published:** 2026-06-07

**Authors:** Andrea Chiara Sansotera, Chiara Ceresa, Cesar Francisco Trejo, Alex Ferrandi, Gianna Allegrone, Silvio Aprile, Maurizio Rinaldi, Silvia Morel, Letizia Fracchia

**Affiliations:** 1 Department of Pharmaceutical Sciences, Università del Piemonte Orientale “A. Avogadro”, 28100 Novara, Italy; andrea.sansotera@uniupo.it (A.C.S.); chiara.ceresa@uniupo.it (C.C.); alex.ferrandi@gmail.com (A.F.); gianna.allegrone@gmail.com (G.A.); silvio.aprile@uniupo.it (S.A.); maurizio.rinaldi@uniupo.it (M.R.); 2 ROELMI BIOTECH Srl, 21040 Origgio, Italy; cesar.trejo@roelmibiotech.com; 3 Novanalitica Srls, 28100 Novara, Italy

**Keywords:** agro-wastes, *Bacillus subtilis*, biosurfactant, fermentation, lipopeptide, Plackett–Burman experimental design, rice husk, surfactin, sustainable production

## Abstract

Nowadays, approximately 50% of chemical surfactants come from petrochemical sources and pose environmental risks due to poor biodegradability, affecting microbial communities, aquatic organisms, and terrestrial ecosystems. Biosurfactants are eco-friendly alternatives, thanks to their strong surface tension-reducing activity, stability, low toxicity, and biodegradability, but their large-scale production is still limited by high costs and low yields. In this study, rice husk was evaluated as a renewable substrate from the agro-industrial field for lipopeptide production by the endophytic *Bacillus subtilis* AC7. Medium optimization through Plackett–Burman designs identified nitrogen sources and pH 6.5 as key factors enhancing biosurfactant production. Under optimized conditions, surfactin production increased from 4.2 mg/L in untreated rice husk to 266–276 mg/L with NaNO_3_ and NH_4_NO_3_ supplementation, respectively. Combined laccase–amylolytic pretreatment further improved substrate utilization, enhancing sugar availability and supporting higher biomass and metabolic activity. In bench-scale fermentation, this condition yielded the highest surfactin concentration (237.5 mg/L). LC-MS/MS analysis confirmed surfactin as the main product, with C15 as the dominant homologue, in both shake-flask and bench-scale fermentations. These findings highlight a novel, sustainable process for surfactin production, offering a renewable alternative to synthetic surfactants while addressing both environmental and economic concerns.

## 1. Introduction

Chemical surfactants are essential components in a wide range of everyday products and industries, including agriculture, detergency, food processing, healthcare, and cosmetics. Despite their extensive applications, approximately 50% of them are derived from petrochemical sources and pose significant environmental risks [1,2]. It has been estimated that nearly 60% of the annual global usage (over 15 million tonnes) of chemical surfactants enters aquatic environments and soils, adversely affecting microbial communities, aquatic and terrestrial organisms, and wastewater treatment processes [3,4]. In this context, biosurfactants (BSs) are increasingly recognized as environmentally friendly, biobased alternatives to synthetic surfactants [3,5]. Compared to their chemical counterparts, they offer distinct advantages, such as low toxicity, high selectivity and biodegradability, lower critical micellar concentration (CMC), and stability under extreme conditions of temperature, pH, and salinity [6,7,8].

BSs are amphiphilic molecules synthesized by microorganisms, including genera such as *Pseudomonas*, *Bacillus*, *Corynebacterium*, and *Rhodococcus* [9]. In particular, lipopeptides produced by *Bacillus subtilis* are of considerable interest due to their exceptional surface activity, stability, and broad spectrum of biological activities [8,10].

Despite these advantages, their large-scale production remains challenging due to limited availability and high costs, which are mainly associated with downstream processing and can account for 60–80% of total production cost and energy demand [11,12,13,14]. Strategies such as the selection of novel producing microorganisms, alongside the improvement of fermentation, extraction, and purification techniques, can increase their commercial potential [15,16].

These advances in microbial production and processing can be further harnessed by utilizing abundant agricultural residues as renewable substrates to produce biomass, enzymes, metabolites, and bioactive compounds [17].

In recent years, increasing attention has been directed toward the use of renewable, low-cost substrates derived from lignocellulosic biomass and agro-industrial residues as a strategy to improve the economic feasibility of biosurfactant production. Feedstocks such as agricultural straw, sugarcane bagasse, molasses, and waste cooking oils have been widely investigated as alternative carbon sources, demonstrating the potential to reduce production costs while contributing to waste valorization and circular bioeconomy frameworks [16,18,19,20,21]. These approaches not only lower raw material costs but also mitigate environmental impacts associated with waste disposal.

This opportunity has been amplified by the global increase in rice production, thanks to advanced agricultural practices, resulting in a significant accumulation of rice processing residues [22]. Italy, the leading rice producer in Europe, cultivates rice over roughly 220,000 hectares, primarily in the lower Po Valley and a narrow belt extending towards the Pre-Alps in Lombardy and Piedmont. These areas benefit from abundant water resources, facilitating extensive irrigation. Notably, the provinces of Vercelli, Pavia, Novara, and Milano account for 90% of national rice production and are the main sources of rice husk residues [23].

Rice husk, the fibrous outer layer (hull) of the rice grain, makes up approximately 18–25% of the paddy weight. It is composed predominantly of lignocellulosic material (cellulose, hemicellulose, and lignin), substantial amounts of silica and moisture, and small proportions of proteins, lipids, and mineral trace elements [19,24]. This composition classifies it as a cost-efficient, valuable resource, aligning with the tenets of bioeconomics and highlighting the synergy between sustainable development aims and the exploitation of food waste [19,25].

However, due to its structural complexity, an efficient microbial utilization of rice husk remains a critical challenge [18,19]. To overcome these limitations, various pretreatment strategies have been explored to convert these complex polymers into simpler, fermentable sugars. Among them, enzymatic pretreatment has gained increasing interest due to its specificity, mild operating conditions, and reduced formation of inhibitory by-products compared to chemical or physical methods [26].

Several studies on biosurfactant production from agro-industrial residues have focused on the use of these materials as a carbon source [27,28,29,30,31]. In the specific case of rice husk, previous studies have mainly investigated its use in combination with other substrates or as a structural support in solid-state fermentation systems, not focusing specifically on the optimization of a liquid fermentation medium based on rice husk and its enzymatic hydrolysates [32,33,34,35]. Furthermore, a knowledge gap exists regarding the development of integrated bioprocess strategies that combine lignocellulosic substrate valorization with targeted pretreatment and fermentation optimization to enhance lipopeptide production.

In this context, the present study aims to investigate surfactin production by *Bacillus subtilis* AC7 using rice husk as the primary substrate, integrating statistical experimental design, enzymatic pretreatment, and both shake-flask and bench-scale fermentation approaches. Unlike previous studies, this work evaluates rice husk not only as a low-cost supplementary substrate or bulking agent but as a central component of the fermentation medium, while also assessing the impact of enzymatic hydrolysis on substrate accessibility, microbial growth, and biosurfactant production. By combining process optimization with substrate valorization, this study proposes a novel and sustainable platform for lipopeptide production, contributing to the production of economically viable and environmentally friendly alternatives to synthetic surfactants.

## 2. Materials and Methods

### 2.1. Bacterial Strain and Maintenance

The lipopeptide-producing endophytic strain *B. subtilis* AC7 was originally isolated from the inner stem tissue of *Robinia pseudoacacia* and identified by complete 16S rDNA sequence analysis [36]. The strain was stored at −80 °C in Luria–Bertani (LB) broth (Scharlab, Barcelona, Spain) supplemented with glycerol (Scharlab, Spain). For routine maintenance, the strain was cultivated either on LB agar (Scharlab, Spain) at 28 °C for 24 h or in LB broth at 28 °C for 24 h at 120 rpm.

### 2.2. Rice Husk Media Preparation and B. subtilis AC7 Cultivation for Lipopeptide Production

Rice husk was kindly provided by a local rice producer in the Vercelli province, Piedmont region (Azienda Agricola Deambrogio Franco, Tenuta Val Serpe, 13046 Lamporo, Italy). Its chemical composition was analyzed by the chemistry laboratory of the Chamber of Commerce of Turin (Table 1). The rice husk was milled for 3 min using a food homogenizer (“La Moulinette D56”, Mulinex, Barcelona, Spain) to obtain finely ground and homogenous particles, which were then suspended in deionized water at a concentration of 25 g/L and mixed with a magnetic stirrer for 10 min.

The rice husk medium was subsequently supplemented with definite constituents and pH adjusted to either 6.5 or 7.5, according to the experimental designs described in Table 2 and Table 3 (Section Experimental Design for the Identification of Critical Medium Components for Lipopeptide Production in Rice Husk). Media were sterilized by autoclaving at 121 °C for 15 min.

*B. subtilis* AC7 was cultivated in these media at 28 °C for 24 h and 48 h. Cultures were performed in 1 L Erlenmeyer flasks containing 250 mL of culture medium and inoculated with 2% (*v*/*v*) of a seed culture prepared under the same conditions. Incubation was carried out on a rotary shaker at 140 rpm. At defined time points (0 h, 24 h, and 48 h), culture samples were collected, centrifuged, and the surface tension of cell-free samples was measured by the Du Noüy ring method with a KSV Sigma 703D tensiometer (KSV Instruments Ltd., Espoo, Finland) to detect the presence of lipopeptides. Four independent measurements were performed for each sample (*n* = 4).

#### Experimental Design for the Identification of Critical Medium Components for Lipopeptide Production in Rice Husk

To evaluate the effects of variables on surface tension with a limited number of experiments, a Plackett–Burman (PB) experimental design with 6 two-level factors and 12 runs was implemented. The resulting experimental layout is shown in Table 2.

To explore the influence of other factors, a second PB experimental design with 7 two-level factors and 12 runs was generated (Table 3).

For both designs, surface tension (T) was measured at 0 h, 24 h, and 48 h for each experimental condition. The response variable was defined as the difference in surface tension (ΔT) at 24 h and 48 h, with respect to time 0 h.

For each experimental design, a linear model was constructed using the design variables (X) and time (t) as predictors, with ΔT as the outcome. The main effect (*m_x_*) of each variable X was computed as:

(1) $$m_{x}=\frac{1}{2n_{+}}\sum {\Delta T}_{+1}-\frac{1}{2n_{-}}\sum {\Delta T}_{-1}$$

where *n_+_* and *n*_−_ represent the number of experiments at higher and lower levels, respectively, and ΔT_+1_ and ΔT_−1_ are the corresponding observed values.

For each PB design, a linear model was fitted using least squares regression, and the significance of each factor was evaluated through analysis of variance (ANOVA).

### 2.3. Viable Counts, pH, and Surface Tension of B. subtilis AC7 Grown in Rice Husk-Based Media

To characterize growth dynamics, pH variation, and lipopeptide production, *B. subtilis* AC7 was cultivated in four rice husk-based media (25 g/L), which were supplemented with different inorganic salts, selected based on the PB experimental design outcomes: NaNO_3_ (3 g/L), NH_4_NO_3_ (3 g/L), (NH4)_2_SO_4_ (2.5 g/L), and MgSO_4_ (1 g/L). Basal rice husk medium without supplementation was used as a control. The initial pH of all media was adjusted to 6.5, and sterilization was performed by autoclaving at 121 °C for 15 min.

*B. subtilis* AC7 was thawed and streaked on rice husk agar plates (25 g/L rice husk and 15 g/L bacteriological agar) and incubated at 28 °C for 24 h. A single colony was then transferred into 5 mL of physiological saline (0.9% *w*/*v* NaCl) and incubated at 28 °C with shaking at 120 rpm for 2 h to obtain a homogeneous suspension (pre-inoculum). When the OD_600_ reached approximately 0.5, 500 µL of the pre-inoculum was used to inoculate 250 mL of the rice husk-based media.

Cultures were incubated at 28 °C for 24, 48, and 144 h to monitor the complete growth curves up to the stationary phase. At each time point, samples were collected for viable cell counts, pH, and surface tension measurements. Viable counts were determined by serial dilution and plating on rice husk agar. Plates were incubated at 28 °C for 24 h, and colonies were counted under a stereomicroscope (Nikon SMZ200, Tokyo, Japan) to calculate colony-forming units per milliliter (CFU/mL). Three measurements were performed for each sample (*n* = 3).

### 2.4. Batch Production and Extraction of Lipopeptide AC7 in Selected Media

For extraction yield calculation and chemical characterization of lipopeptide AC7, *B. subtilis* AC7 was grown in LB broth and in different rice husk media under selected culture conditions: Sample 1 (rice husk, 48 h growth); Sample 2 (rice husk + (NH_4_)_2_SO_4_ 2.5 g/L and NaNO_3_ 3 g/L, 48 h growth); Sample 3 (rice husk + (NH_4_)_2_SO_4_ 2.5 g/L, MgSO_4_ 1 g/L, NaNO_3_ 3 g/L, NH_4_NO_3_ 3 g/L, 48 h growth); Sample 4 (NaNO_3_ 3 g/L, 48 h growth); and Sample 5 (NH_4_NO_3_ 3 g/L, 48 h growth). Growth was carried out for 48 h. For lipopeptide extraction, culture broths were centrifuged at 6000 rpm for 20 min at 4 °C to remove microbial cells and rice husk residues. The extraction procedure followed the method described by Ceresa et al. [40]. Briefly, cell-free supernatants were acidified to pH 2.2 with 6 M HCl and incubated overnight at 4 °C. The precipitated lipopeptide was extracted three times with ethyl acetate–methanol (4:1, *v*/*v*; Sigma–Aldrich, Darmstadt, Germany). The organic phases were dehydrated, filtered, and evaporated under vacuum. The resulting lipopeptide AC7 was dissolved in acetone (Sigma–Aldrich, Germany), transferred to pre-weighed glass tubes, and re-evaporated to remove the solvent. The dry residue was then weighed to calculate the extraction yield, expressed as mg/L.

### 2.5. Chemical Characterization of Lipopeptide AC7 Produced in Batch Tests on Rice Husk Media

#### 2.5.1. Reference Standard Sample Preparation

Surfactin, which was used as the reference standard for quantitative analysis, was purified from crude extract following the method reported by Pecci et al. [41], with modifications.

The biosurfactant AC7 (AC7BS), extracted from *B. subtilis* AC7 cultured in LB broth, was subjected to chromatography on silica gel (230–400 mesh; Merck KGa, Darmstadt, Germany). Elution was first carried out with chloroform, followed by a chloroform/methanol mixture with a stepwise increase in methanol from 0 to 40%. The process was monitored by thin-layer chromatography (TLC) carried out on pre-coated silica gel 60 F254 plates (Merck Co. Inc, Damstadt, Germany). TLC plates, spotted with the purified biosurfactant samples dissolved in methanol, were developed with methanol/chloroform/acetic acid (89:9:2, *v*/*v*/*v*) as the mobile phase, and visualized by spraying plates with non-specific reagent (3% KMnO_4_).

Among the five collected fractions, one major fraction contained surfactin homologues. This purified fraction (surfactin standard) and the crude biosurfactant extracts were subsequently analyzed by liquid chromatography–tandem mass spectrometry (LC-MS/MS) to determine surfactin content. Each homologue was quantified according to the relative peak area percentage.

For LC-MS/MS analysis, aliquots of the crude extracts or purified fraction were dissolved in methanol/acetonitrile (50/50 *v*/*v*) to obtain a 1 mg/mL stock solution. Working standard solutions containing the reference fraction were prepared by diluting the stock solution with methanol to appropriate concentrations. Both stock and working solutions were stored at 4 °C.

#### 2.5.2. Mass Spectrometry Analysis

Analyses were performed on a Surveyor HPLC system coupled to an LCQ DECA XP Plus Ion Trap mass spectrometer (Thermo Finnigan, LC and LC/MS Division, San José, CA, USA) equipped with an electrospray ionization (ESI) source.

Separation was obtained by reversed-phase chromatography on a Luna 5 µm C18 column (150 × 2.0 mm, Phenomenex Inc., Torrance, CA, USA) protected with a C18-Security Guard cartridge (4 × 2.0 mm: Phenomenex Inc, Torrance, CA, USA), maintained at 30 °C. The mobile phase consisted of 10 mM of ammonium formate buffer (pH 5.2, solvent A) and acetonitrile (solvent B), and the flow rate was 0.4 mL/min. The gradient, initially set to 40% B from 0 to 4 min, was increased to 100% B from 4 to 24 min, remained at 100% B from 24 to 30 min, and was then decreased to 40% over 5 min and held constant for equilibration. The total run time was 40 min, and the injection volume was 5 µL.

The mass spectrometer was equipped with an ESI source. The electrospray ionization–mass spectrometry (ESI-MS) and electrospray ionization–tandem mass spectrometry (ESI-MS/MS) spectra of the biosurfactant congeners were recorded using direct infusion of sample solutions, and the optimal values for ESI source parameters were determined as follows: the source voltage and capillary voltage were at 4.80 kV and 23 V, respectively, in positive mode. The capillary temperature was maintained at 350 °C, and nitrogen was used as the nebulizing gas at 30 arbitrary units. Data were acquired in positive ion mode in full-scan MS (*m*/*z* 100–2000) and MS/MS product ion scan. Normalized collision energy (NCE %) was optimized for each precursor ion: *m*/*z* 1030.5 (36%), 1044.5 (37%), 1058.5 (37%), 1478 (35%), and 1506 (35%) [36].

For multiple reaction monitoring (MRM) in LC-MS/MS analysis, precursor ions [M+Na]^+^ were selected according to the conditions set during the infusion experiments. Data were acquired in positive modality, and transitions used for identification were: C13-surfactin: *m*/*z* 1030.5 → 707, 786 (NCE 36%); C14-surfactin: *m*/*z* 1044.5 → 707, 800 (NCE 36%); and C15-surfactin: *m*/*z* 1058.5 → 707, 814 (NCE 38%).

#### 2.5.3. Surfactin Quantification

Surfactin homologues (C13, C14, and C15) were individually quantified in samples by analysis of the purified fraction used as the reference standard. Each calibration curve was constructed using seven concentration levels of surfactin standard solutions, each injected in triplicate. For each species, limits of detection (LOD) and quantification (LOQ) were estimated using the slope (S) and the standard deviation of the response (σ) according to the formulas LOD = (3.3 × σ)/S and LOQ = (10 × σ)/S.

### 2.6. Optimization of Rice Husk Medium by Enzymatic Pretreatment

For the optimization experiments involving enzymatic pretreatments, rice husk-based media were prepared from clarified rice husk extracts or hydrolysates obtained after thermal and/or enzymatic treatment, followed by removal of solid residues by filtration. This approach was adopted to ensure comparability among treatments and to facilitate subsequent scale-up applications.

#### 2.6.1. Laccase Pretreatment

Milled rice husk was pretreated with the recombinant laccase enzyme rPOXA1b [42], kindly provided by Roelmi HPC (Origgio, Italy) in collaboration with BioPox s.r.l. (Naples, Italy).

Rice husk was suspended in 0.5 M sodium citrate buffer (pH 5.0) at a concentration of 2.5% (*w*/*v*) in Erlenmeyer flasks. The suspensions were mixed with a magnetic stirrer and heat-treated at 70 °C for 1 h. After cooling, laccase was added at concentrations ranging from 2 to 100 U/g of dry substrate. Flasks were incubated at 28 °C for 24 h under shaking (150 rpm). Following incubation, the enzyme was inactivated by autoclaving at 121 °C for 15 min. The suspensions were then filtered through a coarse mesh sieve, centrifuged at 5000 rpm for 15 min to collect the rice husk residues, and subsequently filtered through 0.22 μm membrane filters. The resulting filtrate was supplemented with NaNO_3_ (3 g/L) and adjusted to pH 6.5. A second sterilization step was performed by autoclaving at 121 °C for 15 min prior to use as a culture medium.

#### 2.6.2. Alpha-Amylase and Glucoamylase Pretreatment (RHH)

For RHH preparation, the hydrolysis of rice husk was carried out in two stages: liquefaction and saccharification, using a 2 L Erlenmeyer flask with a working volume of 1 L. The protocol was performed following established methodologies [43,44,45,46] with slight modifications.

A 2.5% (*w*/*v*) rice husk suspension was prepared in an Erlenmeyer flask in phosphate buffer, and the pH was set at 6.1. Liquefaction was conducted at 80 °C for 30 min, using α-amylase (MP Biomedicals, ≥700 u/mg protein) at a dosage of 0.03% of the weight of starch contained in the rice husk. After a subsequent heating phase in a water bath at 70 °C for 30 min at pH 4.8, the enzyme was deactivated by heating at 90 °C for 10 min. Following this step, the sample was allowed to cool and maintained at 55 °C. For the saccharification phase, glucoamylase (TCI chemicals) was added at a concentration of 0.02 U/mg of starch contained in the rice husk. After incubation at 37 °C for 24 h, the suspension was filtered to remove rice husk particles, which were supplemented with NaNO_3_ (3 g/L) and sterilized at 121 °C for 15 min. The final pH of the medium was adjusted to 6.5.

#### 2.6.3. Laccase, α-Amylase, and Glucoamylase Pretreatments (RHH-LAC)

The RHH-LAC media were prepared by subjecting rice husk to the laccase pretreatment described in Section 2.6.1, followed by starch hydrolysis as detailed in Section 2.6.2. Briefly, rice husk was treated with the recombinant laccase rPOXA1b at concentrations of 60, 80, or 100 U/g of dry substrate and subsequently subjected to liquefaction with α-amylase and saccharification with glucoamylase, as performed for RHH. The resulting hydrolysates were filtered, supplemented with NaNO_3_ (3 g/L), and adjusted to pH 6.5. Media were sterilized at 121 °C for 15 min prior to use.

#### 2.6.4. Control Rice Husk Medium (RH)

A control rice husk medium was prepared using a procedure comparable to that adopted for the enzymatically treated media. Briefly, rice husk (2.5% *w*/*v*) was suspended in water, heated at 70 °C for 2 h under magnetic stirring, and subsequently filtered to remove rice husk particles. The resulting filtrate was supplemented with NaNO_3_ (3 g/L), sterilized at 121 °C for 15 min, and then adjusted to pH 6.5 prior to use.

### 2.7. Evaluation of B. subtilis AC7 Growth in Enzyme-Treated Rice Husk Media Using the BioTek Cytation 5 System

The growth of *B. subtilis* AC7 in media containing enzymatically treated rice husk, prepared as described in Section 2.6.1, Section 2.6.3 and Section 2.6.4, was evaluated using the automated plate reader BioTek Cytation 5 (Agilent Technologies, Winooski, VT, USA), which allowed continuous monitoring under controlled conditions. Twenty-four-well plates containing 800 μL of each medium were inoculated in duplicate with 2% (*v*/*v*) of a pre-culture and incubated at 28 °C with orbital shaking at 140 rpm for 15 h. Optical density at 600 nm (OD_600_) was recorded every 20 min throughout incubation. The experiment was performed in duplicate on two different days (*n* = 4).

### 2.8. Evaluation of B. subtilis AC7 Growth in Enzyme-Treated Rice Husk Media in Batch Tests

The growth of *B. subtilis* AC7 in enzyme-treated rice husk media was evaluated under shake-flask conditions. Cultures were prepared in 1 L Erlenmeyer flasks containing 200 mL of RH, RHH, or RHH-LAC (prepared with 100 U/g laccase). Each flask was inoculated with 2% (*v*/*v*) pre-inoculum and incubated at 28 °C with shaking at 140 rpm for 72 h.

Samples were collected at 0, 6, 24, 48, and 72 h to evaluate cell growth by OD_600_, pH, reducing sugar consumption (*n* = 3), and surface tension (*n* = 6).

#### Quantification of Reducing Sugars During Fermentation Using the 3,5-Dinitrosalicylic Acid (DNS) Method

Reducing sugars were quantified using the 3,5-dinitrosalicylic acid (DNS) method [47], with slight modifications, on samples collected during flask and bioreactor fermentations to monitor sugar consumption over time. Aliquots were withdrawn at 0, 6, 24, 48, and 72 h and centrifuged to remove cells. Supernatant (1 mL) was mixed with 1 mL of DNS reagent, boiled for 10 min until a red–orange color developed, cooled, and brought to a final volume of 10 mL with distilled water. Absorbance was measured at 540 nm using a UV–Vis spectrophotometer. A glucose calibration curve (0.1–1.0 g/L) was prepared in parallel, processed under the same conditions, and the results were expressed as g/L glucose equivalents.

### 2.9. Lipopeptide AC7 Production in a Bench-Scale Bioreactor

To assess the potential for enhancing the efficiency of lipopeptide AC7 production on the optimized rice husk medium, experiments were conducted in a bench-scale bioreactor setting using both untreated rice husk and rice husk hydrolyzed with α-amylase and glucoamylase.

#### 2.9.1. Inoculum Preparation and Cultivation in a Bench-Scale Bioreactor

A *B. subtilis* AC7 seed culture was incubated in LB broth for 24 h at 28 °C with agitation at 140 rpm using an ES-20/80C orbital incubator shaker (Biosan, Riga, Latvia). Subsequently, the seed culture at OD_600_ = 0.3 (corresponding to approximately 10^8^ CFU/mL) was used to inoculate RH, RHH, and RHH-LAC media at a concentration of 2% (*v*/*v*) for lipopeptide AC7 production. Batch fermentations were performed in a 1 L bioreactor (Applikon miniBio, Getinge, Göteborg, Sweden) with a working volume of 0.8 L. The fermentations were carried out for 72 h, and the pH was maintained at 6.5 using a peristaltic pump to deliver 1 M of NaOH. The fermentation temperature was maintained at 28 °C, while agitation, provided by two Rushton impellers, was gradually increased from 200 to 500 rpm. A continuous airflow of 2 L/min was supplied, and the bioreactor was aerated through a sparger. A silicone-based antifoam (silicone antifoam 15%; VWR, Avantor Science, Melville, NY, USA) was used to manage foam formation. Under these conditions, the percentage of dissolved oxygen in the medium was 100% during fermentation. The fermentation process was monitored and controlled using the Lucullus^®^ PIMS system 3.05 (Securecell, Urdorf, Switzerland). Bacterial growth was monitored by measuring the turbidity of the cultures at OD_600_ and the reducing sugar consumption at 0, 6, 24, 48, and 72 h (*n* = 3). Surface tension was measured after 72 h (*n* = 6).

#### 2.9.2. Chemical Characterization of Lipopeptide AC7 Extracted from Bench-Scale Bioreactor Cultures

Lipopeptide extraction and chemical characterization from the supernatant of bench-scale bioreactor fermentations followed the procedure outlined in Section 2.5.

### 2.10. Statistical Analysis

All statistical analyses and graphical representations were performed using R software version 4.5.3 (11 March 2026) [48].

## 3. Results

### 3.1. Identification of Critical Medium Components for Lipopeptide Production in Rice Husk

Two Plackett–Burman (PB) experimental designs were used to identify the most significant factors influencing lipopeptide production by *B. subtilis* AC7 grown on rice husk medium. For this purpose, respectively, six and seven independent variables and their respective high and low concentrations or values were chosen in experimental designs 1 and 2 (Table 2 and Table 3, Section 2). The selection of variables and their corresponding levels was based on data available in the literature.

The increase in surface tension relative to time 0 (ΔT) was modeled using the following equation:

(2) $$\Delta T=\beta_{0}+\sum_{i=1}^{n}\beta_{i}X_{i}+\beta_{t}t$$

where ΔT represents the change in surface tension with respect to the baseline (T_t_ − T_0_), X_i_ denotes the six (or seven) design variables, and t (time) is treated as a categorical factor with two levels (24 h and 48 h). The coefficients β_i_ and β_t_ represent half of the main effects of the corresponding variables. All variables, including time, were coded as ±1.

Table 4 and Table 5 show the ΔT of cell-free supernatants of *B. subtilis* AC7 in the two PB experimental designs for t = 24 h and t = 48 h.

Coefficient estimates and *p*-values of the two PB designs are reported in Table 6, where negative coefficients (i.e., those corresponding to factors related to the decrease in surface tension) are highlighted in bold.

**Figure 1:**
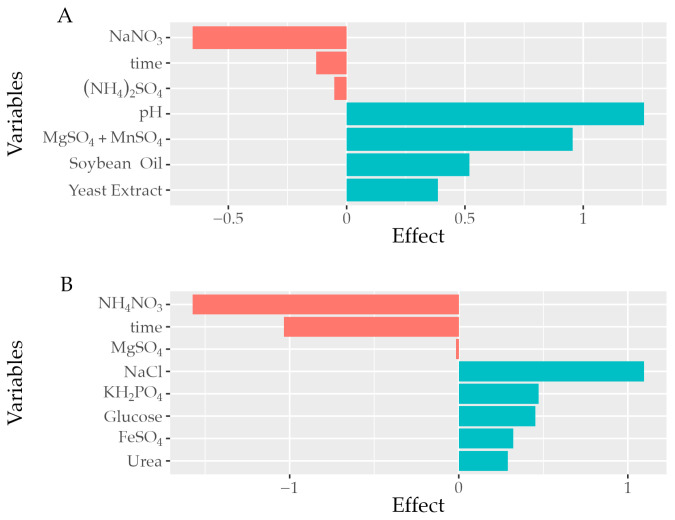
The main effects of each variable considered in the two PB experimental designs applied for the optimization of lipopeptide AC7 production in rice husk medium. Time was not part of the original PB design but was included in the regression model as an additional categorical factor (24 h and 48 h); its estimated effect is therefore shown in the plot. (**A**) Variables and corresponding effects in the first PB experimental design. (**B**) Variables and related effects in the second PB experimental design.

In addition, the estimates of the main effects of each factor are presented in Figure 1A,B.

In the first PB experimental design (Figure 1A), NaNO_3_ supplementation emerged as the variable most positively associated with surface tension reduction, followed by incubation time and (NH_4_)_2_SO_4_, although none of these effects reached statistical significance. In contrast, pH increased to 7.5, and supplementation with MgSO_4_+MnSO_4_, yeast extract, or soybean oil correlated negatively with ΔT, suggesting reduced lipopeptide synthesis.

In the second PB experimental design (Figure 1B), adjustments were made to the variables and their levels based on the literature data, as well as to align with the results obtained in the initial design. Specifically, NaNO_3_ was replaced with NH_4_NO_3_, which showed a strong and significant positive correlation with surface tension reduction (*p* = 0.005), confirming the importance of the nitrogen source. Time also maintained a significant positive effect (*p* = 0.048). Separating Mg^2+^ and Mn^2+^ ions revealed a weak but non-significant association with ΔT, whereas KH_2_PO_4_, NaCl, FeSO_4_, glucose, and urea showed a negative correlation with surface tension decrease. Notably, NaCl exerted a significant adverse effect on surface tension reduction (*p* = 0.037).

Through experimental design optimization, culture conditions favorable for lipopeptide AC7 production in rice husk medium were identified. Specifically, the addition of NaNO_3_ and NH_4_NO_3_ to rice husk broth was associated with the greatest reduction in surface tension, although the effect of NaNO_3_ was not statistically significant. Overall, these designs highlighted that the presence of a nitrogen source, an incubation time of 48 h, and a pH of 6.5 were the most critical determinants for surface tension reduction and, by inference, biosurfactant production in rice husk broth.

### 3.2. Growth Kinetics in Rice Husk Medium, pH Dynamics, and Surface Tension Reduction

**Figure 2:**
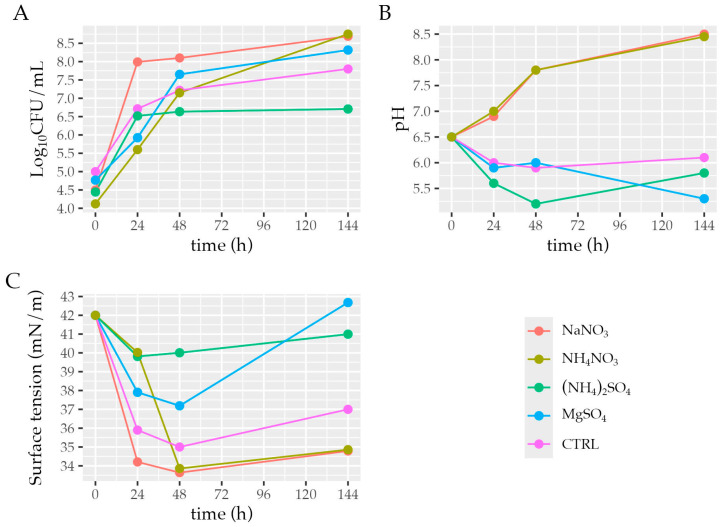
Growth dynamics, pH variation, and surface tension changes in *B. subtilis* AC7 cultured in rice husk medium supplemented with different salts. (**A**) Growth curves expressed as Log_10_ CFU/mL over 144 h. (**B**) Culture broth pH values measured at the same intervals; (**C**) surface tension of cell-free supernatants, measured as an indirect indicator of biosurfactant production.

To further investigate the results obtained from the Plackett–Burman screening, growth dynamics, pH variation, and surface tension reduction were further evaluated in selected rice husk-based media up to 144 h, in order to monitor the complete growth profile until the stationary phase. Media composition and cultivation parameters were selected based on factors associated with a reduction in surface tension (i.e., negative estimated effects), independently of their statistical significance. Four rice husk-based media (25 g/L) supplemented respectively with NaNO_3_ (3 g/L), NH_4_NO_3_ (3 g/L), (NH_4_)_2_SO_4_ (2.5 g/L), and MgSO_4_ (1 g/L) were inoculated with *B. subtilis* AC7, and then incubated at 28 °C. The supernatant surface tension, pH, and viable counts were measured at different time intervals (24, 48, and 144 h) and compared to the unsupplemented control (CTRL) (Figure 2).

Growth was expressed as viable counts (Log_10_ CFU/mL) over a 144 h incubation period (Figure 2A). At inoculation (time 0), all conditions started with similar cell densities (about 4.2–5.0 Log_10_ CFU/mL). During the first 24 h, a rapid increase was observed in all media. In particular, NaNO_3_ supplementation showed the highest growth, with cell densities reaching 8.0 Log_10_ CFU/mL and maintaining continuous bacterial growth up to ~8.7 Log_10_ CFU/mL at 144 h.

Although NH_4_NO_3_ exhibited a slower growth dynamic compared to the other salts, the cell density increased, reaching CFU values of ~8.8 Log_10_ CFU/mL, similar to those observed for NaNO_3_. MgSO_4_ and the CTRL medium showed intermediate growth profiles. In contrast, starting from 24 h, (NH_4_)_2_SO_4_ resulted in cell densities below those of the other media, with growth stabilizing around 6.6 Log_10_ CFU/mL.

The differences observed in growth dynamics were also confirmed by pH measurements obtained during microbial growth (Figure 2B). All media were initially adjusted to pH 6.5, a value that previously emerged as positively correlated to biosurfactant production.

As shown in Figure 2B, a clear difference between the media supplemented with NaNO_3_ and NH_4_NO_3_ compared to the other salts and the control was already noticeable within the first 24 h. For the two nitrates, a rapid increase in pH was observed, reaching values of around 7.8 in the first 48 h and rising further to around 8.5 at 144 h. In contrast, all other salts and the control showed media acidification to values around or slightly below 6, with the pH falling to 5.2 at 48 h for (NH_4_)_2_SO_4_.

Surface tension measurements of the cell-free supernatants suggested biosurfactant production by *B. subtilis* AC7 in the CTRL medium and in media supplemented with different salts (Figure 2C). All cultures started from an initial value of 42 mN/m. In particular, NaNO_3_ supplementation led to the strongest reduction, reaching 34.2 mN/m after 24 h and 33.6 mN/m at 48 h, confirming its efficiency in promoting lipopeptide production. Similarly, a good reduction was observed with NH_4_NO_3_ at 48 h (34.0 mN/m), closely followed by the CTRL medium at 48 h (35.0 mN/m). MgSO_4_ showed intermediate reductions at 24–48 h (around 37–38 mN/m). In contrast, (NH_4_)_2_SO_4_ caused a limited decrease at 24 h, stabilizing around 39–40 mN/m throughout the experiment. At 144 h, surface tension slightly increased in all media, most notably in MgSO_4_, which slightly exceeded the initial value.

### 3.3. Surfactin Detection and Method Development

Preliminary mass spectrometry analyses by direct infusion, following the approach of Ceresa et al. [36], indicated that surfactin is the predominant lipopeptide produced by *B. subtilis* AC7, while fengycin was detected only at low levels. Accordingly, subsequent analyses were focused on surfactin.

An LC-MS/MS method was optimized based on Pecci et al. [41] to allow accurate quantification of surfactin homologues. The stationary phase and mobile phases were identified as the most critical factors influencing resolution and sensitivity. Optimal separation was achieved using a Luna C18 column (150 × 2 mm) with acetonitrile as the organic eluent and ammonium formate buffer as the aqueous phase, providing stable pH conditions during analysis.

Mass spectrometry was carried out in ESI mode, both positive and negative. MS and MS/MS spectra were acquired to characterize surfactin homologues and to optimize the MRM assay for quantification. As expected, surfactins were detected as heterogeneous mixtures differing in fatty acid chain length.

Product ion spectra obtained from ESI–MS/MS experiments were used both for the identification and quantification of the surfactin homologues. The LC-MS/MS method was configured to analyze both purified AC7BS fractions derived from LB medium and crude AC7BS extracts obtained from rice husk media supplemented with different salts (see the following paragraph).

#### 3.3.1. Surfactin Homologue Distribution in LB-Grown Cultures

Table 7 summarizes the MRM experimental conditions established during method development and subsequently applied for quantitative studies. Purified fractions from LB-grown cultures revealed surfactin as a heterogeneous mixture of homologues. The most abundant congener was C15-surfactin ([M+Na]^+^ *m*/*z* 1058.5, Rt 18.62 min), accounting for 70.21% of the total signal, followed by C14-surfactin (18.16%) and C13-surfactin (11.63%).

**Figure 3:**
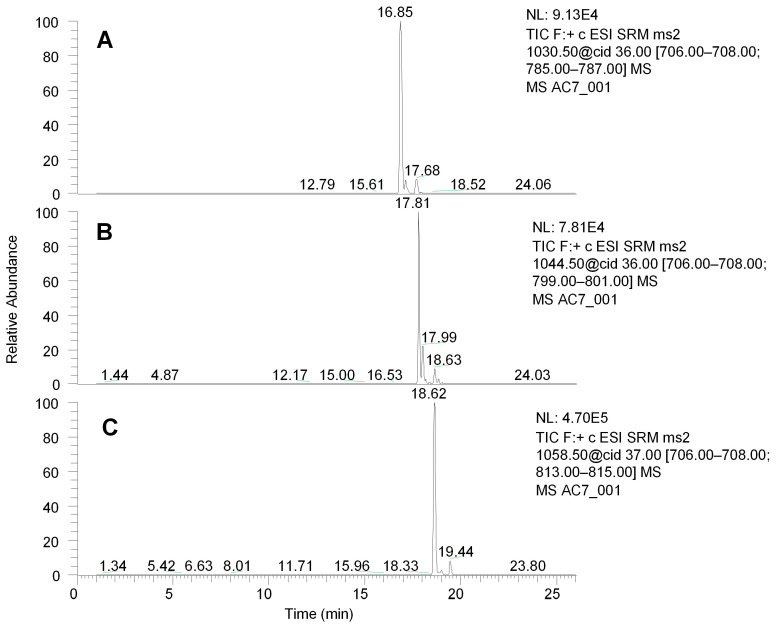
LC-MS/MS mass chromatogram of AC7BS in crude LB extract. Surfactin congeners detected as sodiated ions [M + Na]^+^: (**A**) *m*/*z* 1030 (C13), (**B**) *m*/*z* 1044 (C14), and (**C**) *m*/*z* 1058 (C15).

Figure 3 presents the LC–MS/MS mass chromatogram of the three homologues in crude LB extract. Surfactin homologues were well separated according to the retention times reported in Table 7, with surfactin [M+Na]^+^ ions detected at *m*/*z* 1030 (C13-surfactin, Figure 3A), 1044 (C14-surfactin, Figure 3B), and 1058 (C15-surfactin, Figure 3C).

#### 3.3.2. Surfactin Quantification in AC7BS Extracts from Rice Husk Media

Based on the outcomes of the two PB experimental designs, the most influential factors for lipopeptide production in rice husk medium were identified, with particular emphasis on the role of nitrogen sources (NaNO_3_ in design 1 and NH_4_NO_3_ in design 2). To validate these findings and assess whether the combination of selected variables could exert additive or synergistic effects on biosurfactant synthesis, different rice husk-based formulations were prepared and tested. Specifically, we evaluated (i) rice husk alone, (ii) rice husk supplemented with the most effective variables from design 1, (iii) rice husk supplemented with the optimal variables from both design 1 and design 2, and (iv) rice husk supplemented individually with NaNO_3_ or NH_4_NO_3_. This strategy allowed us to confirm the contribution of individual variables, while also exploring the potential of combined supplementation to enhance surfactin production.

Precisely, the following growth media were employed for *B. subtilis* AC7 growth: Sample LB (LB medium, 48 h growth); Sample 1 (rice husk, 48 h growth); Sample 2 (rice husk + (NH_4_)_2_SO_4_ 2.5 g/L and NaNO_3_ 3 g/L, 48 h growth); Sample 3 (rice husk + (NH_4_)_2_SO_4_ 2.5 g/L, MgSO_4_ 2 g/L, NaNO_3_ 3 g/L, and NH_4_NO_3_ 3 g/L, 48 h growth); Sample 4 (NaNO_3_ 3 g/L, 48 h growth); and Sample 5 (NH_4_NO_3_ 3 g/L, 48 h growth).

Crude lipopeptides were extracted from each growth medium and analyzed by LC-MS/MS. The surfactin calibration curve showed good linearity in the range of 0.5–5 µg/mL, and LOD and LOQ, determined for the most abundant congener C15-surfactin, were fixed at 0.01 µg/mL and 0.4 µg/mL, respectively.

The LC-MS/MS method was then applied to quantify surfactin congeners in crude extracts obtained from *B. subtilis* AC7 cultures grown in rice husk-based substrates with or without supplementation (Samples 1–5).

The production of surfactin by *B. subtilis* AC7 varied markedly depending on the growth medium and culture conditions (Table 8). In LB, the extraction yield reached 382.5 mg/L, with a high purity of 76.1%, corresponding to 290.7 mg/L total surfactin. In contrast, rice husk without supplementation (Sample 1) supported only minimal surfactin formation with 4.19 mg/L (1.6% purity) despite an extraction yield of 262 mg/L, indicating the substrate alone is poorly suited for production. Nitrogen supplementation improved production: in Sample 2, the total surfactin yielded 47.79 mg/L (9.0% purity), while Sample 3 reached 57.76 mg/L (8.0% purity), with extraction yields of 531 and 722 mg/L, respectively. Cultivation in rice husk media supplemented with a single nitrogen source resulted in the highest surfactin levels. Sample 4 yielded 266.4 mg/L (18.0% purity), while Sample 5 produced 275.8 mg/L (16.9% purity) with the largest extraction yield (1632 mg/L).

The analysis of surfactin congener distribution (Table 9) revealed differences between LB and rice husk-based media. In LB medium, C15-surfactin was the predominant homologue, accounting for 72.6% of the total surfactin fraction, whereas C14- and C13-surfactins represented 16.3% and 11.1%, respectively. In contrast, rice husk-based media showed a more balanced congener distribution, with C15-surfactin remaining the most abundant homologue but at lower relative proportions (45.4–47.4%), while C14- and C13-surfactins accounted for approximately 32.6–33.6% and 20.0–22.0%, respectively. Overall, the distribution of surfactin congeners among the different rice husk-based media appeared relatively consistent. These results suggest that growth on rice husk media leads to a slightly more balanced distribution of surfactin homologues compared with LB medium.

### 3.4. Evaluation of B. subtilis AC7 Growth in Enzyme-Treated Rice Husk Media Using the BioTek Cytation 5 System

Due to the lignocellulosic nature of rice husk, an enzymatic delignification pretreatment was carried out using the recombinant laccase rPOXA1b to improve nutrient accessibility for *B. subtilis* AC7. To identify suitable enzyme concentrations, rice husk media pretreated with different concentrations of laccase (2–100 U/g) were inoculated, and bacterial growth was monitored during 15 h of incubation. Growth performance was quantified by calculating the area under the curve (AUC) of OD_600_ values.

**Figure 4:**
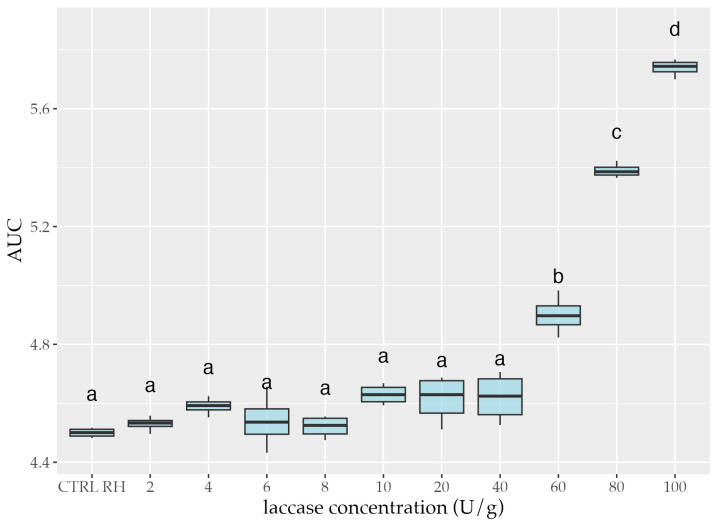
*B. subtilis* AC7 growth in rice husk medium and pretreatment with increasing concentrations of recombinant laccase rPOXA1b (2–100 U/g). Growth was monitored over 15 h. Data are represented as a comparative boxplot illustrating the relationship between the AUC of OD_600_ values and laccase concentration. Different letters indicate significant differences among groups according to Tukey’s post hoc test (*p* < 0.05).

Figure 4 shows a comparative boxplot illustrating the relationship between AUC and laccase concentration. Statistical differences among treatments were assessed by one-way ANOVA followed by Tukey’s post hoc test (*p* < 0.05). A gradual increase in AUC was observed with increasing laccase concentration. Compared to the untreated control (AUC = 4.50 ± 0.02), no significant differences were detected at lower concentrations (2–40 U/g; AUC = 4.53–4.62). In contrast, pretreatment with ≥60 U/g laccase resulted in significantly higher AUC values. The strongest effects on *B. subtilis* AC7 growth were recorded at 80 U/g (AUC = 5.39 ± 0.02) and 100 U/g (AUC = 5.74 ± 0.03) (*p* < 0.0001). These results indicate that enzymatic pretreatment enhanced the growth-supporting capacity of rice husk in a dose-dependent manner. Based on these findings, subsequent experiments focused on laccase concentrations of 60, 80, and 100 U/g.

To further investigate the impact of substrate pretreatment on *B. subtilis* AC7 growth, rice husk-based media were subjected to a two-step enzymatic treatment. The first step consisted of delignification with laccase at 60, 80, and 100 U/g, followed by starch hydrolysis with α-amylase and β-glucoamylase. The control conditions included untreated rice husk (RH) and rice husk subjected only to starch hydrolysis (RHH).

**Figure 5:**
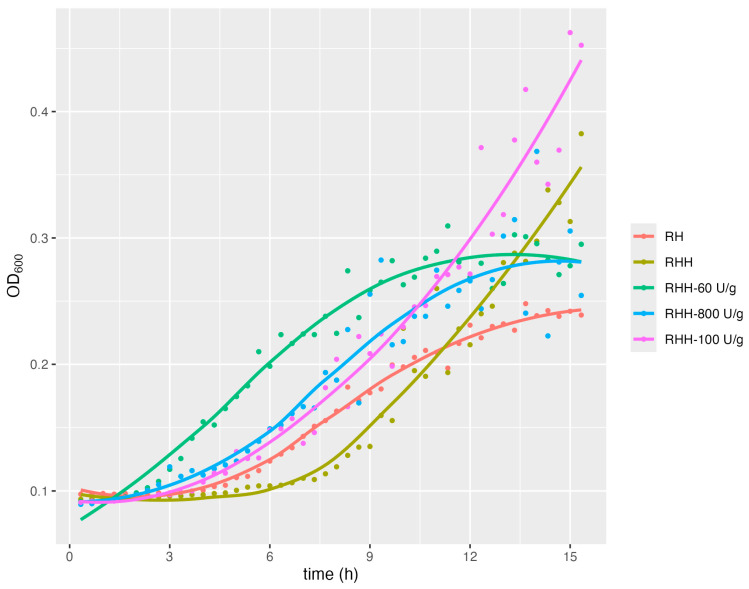
Growth curves of *B. subtilis* AC7 cultivated in 24-well plates in rice husk-based media: untreated (RH), saccharified (RHH), or laccase pretreated (60, 80, or 100 U/g) followed by saccharification (RHH-U/g). Biomass was monitored as optical density at 600 nm (OD_600_) over time (h). Curves were fitted using the Loess method within the R package ggplot2.

Growth was monitored at each condition in 24-well microplates by measuring OD_600_ every 20 min over 15 h using a BioTek Cytation 5 plate reader (Figure 5).

Distinct growth patterns emerged depending on the pretreatment applied. At inoculation (time 0), all cultures started with comparable initial cell densities (OD_600_ about 0.09–0.098). In general, RH, RHH + 60 U/g, and RHH + 80 U/g exhibited relatively short lag phases (about 2 h for RHH + 60 U/g; about 4–5 h for RH and RHH + 80 U/g) and modest biomass accumulation, reaching final OD_600_ values of 0.239–0.295. In contrast, growth in RHH displayed a markedly prolonged lag phase (about 7 h), but once initiated, exponential growth continued throughout the 15 h incubation period, yielding a substantially higher OD_600_ (0.383). The most pronounced effect was observed in RHH + 100 U/g, where the exponential phase started after 4–5 h and persisted beyond 15 h, achieving the highest optical density (0.453), suggesting a significant and further growth enhancement at these conditions.

### 3.5. Evaluation of B. subtilis AC7 Growth in Enzyme-Treated Rice Husk Media in Batch Tests

**Figure 6:**
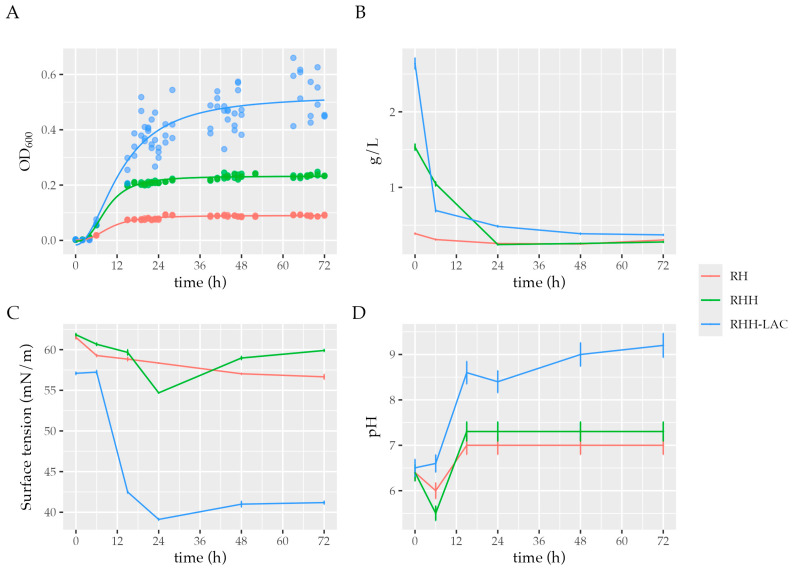
Growth dynamics, sugar consumption, surface tension changes, and pH variation in *B. subtilis* AC7 cultured in rice husk-based media with different enzymatic pretreatments in batch tests. (**A**) Growth curves (OD_600_) in untreated rice husk medium (RH), rice husk subjected to starch hydrolysis (RHH), and rice husk pretreated with both starch hydrolysis and laccase (RHH-LAC). (**B**) Reducing sugar concentrations (expressed as glucose equivalents, g/L) during 72 h growth. (**C**) Surface tension of cell-free supernatants. (**D**) Culture broth pH measured at the same intervals. In panel (**A**), growth curves are constructed using a 4-parameter logistic (4PL) regression model in the R environment, whereas in panels (**B**–**D**), data are presented as mean ± standard deviation connected by segments.

The growth of *B. subtilis* in enzymatic-treated media (RHH and RHH-LAC) was also evaluated in batch tests of up to 72 h by monitoring the OD_600_ of the cultures as well as the content of reducing sugars, surface tension, and pH at different time intervals (0, 6, 24, 48, and 72 h) and comparing to RH (Figure 6).

At inoculation (time 0), all conditions started with similar optical densities (0.002–0.004), and the lag phase ended within 4–6 h (Figure 6A). *B. subtilis* remained in the exponential phase up to 15 h in the case of RH and RHH, and up to 24 h for RHH-LAC, before transitioning to the stationary phase. RHH-LAC supported the highest growth, with an optical density at 72 h of 0.448, compared to RH (0.092), in which a minimal growth of the strain was observed. In RHH, the strain reached an intermediate optical density value of 0.234, suggesting a moderate growth of *B. subtilis* in this medium.

The quantification of reducing sugars (Figure 6B) at time zero confirmed the increased availability of glucose in RHH-LAC (2.6 g/L) and RHH (1.5 g/L) compared to RH (0.4 g/L). Furthermore, both treatments showed a rapid decrease in the initial substrate concentration within the first 24 h. In particular, in RHH-LAC, most of the sugar was rapidly consumed within the first six hours, whereas in RHH, the substrate consumption occurred more gradually within the first 24 h. Conversely, in RH, substrate consumption was minimal, consistent with the limited growth observed in panel A.

Figure 6C,D show the changes in surface tension (mN/m) and pH over time. In both RH and RHH, the surface tension remained relatively stable around 60 mN/m throughout the 72 h period, indicating no significant production of surface-active compounds. Conversely, in RHH-LAC, there was a notable decrease in surface tension, dropping to around 40 mN/m within the first 24 h and remaining low thereafter.

In RH and RHH media, the pH initially slightly dropped within the first 6–12 h and then stabilized around pH 7, remaining relatively constant throughout the 72 h period.

In the RHH-LAC condition, a rapid and significant increase in the pH was observed, reaching values above pH 8 by 24 h and continuing to rise slightly up to 72 h, suggesting the occurrence of an intense metabolic activity that leads to the production of basic by-products.

### 3.6. Scale-Up of Lipopeptide AC7 Production in a Bench-Scale Bioreactor

**Figure 7:**
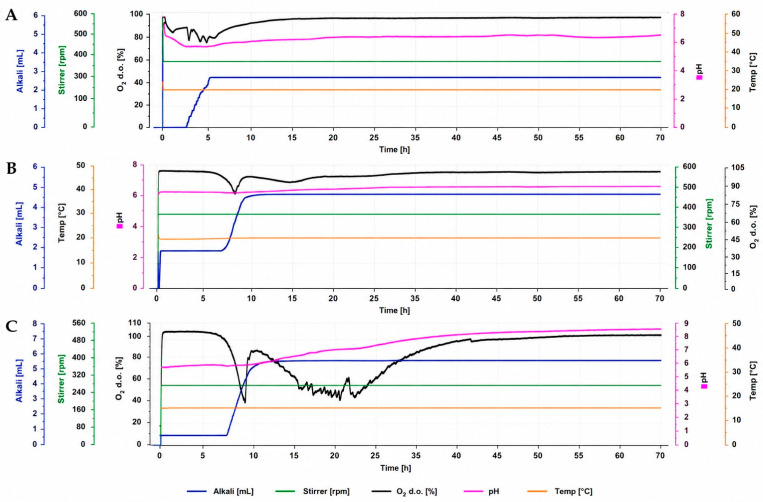
Time-course profiles of bench-scale bioreactor operating parameters during *B. subtilis* AC7 fermentation in (**A**) untreated rice husk (RH), (**B**) hydrolyzed rice husk (RHH), and (**C**) hydrolyzed + laccase-treated rice husk (RHH-LAC). Monitored parameters include dissolved oxygen (DO, %; black line), pH (magenta line), base consumption (mL; blue line), agitation speed (rpm; green line), and temperature (°C; orange line), recorded throughout the 72 h fermentation.

*B. subtilis* AC7 was cultivated in a 1 L bench-scale bioreactor using rice husk-based media subjected to different enzymatic pretreatments (untreated RH, hydrolyzed RH, RHH, and hydrolyzed RH supplemented with laccase (RHH-LAC)) in order to evaluate its metabolic performance under controlled conditions. Continuous monitoring of pH and dissolved oxygen (DO) provided insight into physiological responses and substrate utilization dynamics (Figure 7).

For untreated RH medium (Figure 7A), both pH and DO profiles remained relatively stable throughout the fermentation, indicating limited metabolic activity. A slight initial decrease in DO was followed by recovery to full saturation after approximately 20 h, coinciding with the onset of the stationary phase, consistent with low OD_600_ values and minimal biosurfactant-related activity.

Fermentation in RHH (Figure 7B) showed a moderate metabolic response. DO decreased from approximately 105% to 90% during early growth, reflecting oxygen consumption during the exponential phase, and then stabilized, suggesting reduced metabolic activity after partial substrate depletion. The pH remained within 6.0–7.0, with minor base adjustments.

In contrast, RHH-LAC (Figure 7C) promoted the highest metabolic activity, characterized by a marked DO decrease (to ~55%), strong fluctuations during active growth, progressive alkalinization up to pH 8.0–8.5, and an extended exponential phase. These findings indicate that enzymatic pretreatment, particularly with laccase, improved substrate accessibility and sustained metabolic activity.

**Figure 8:**
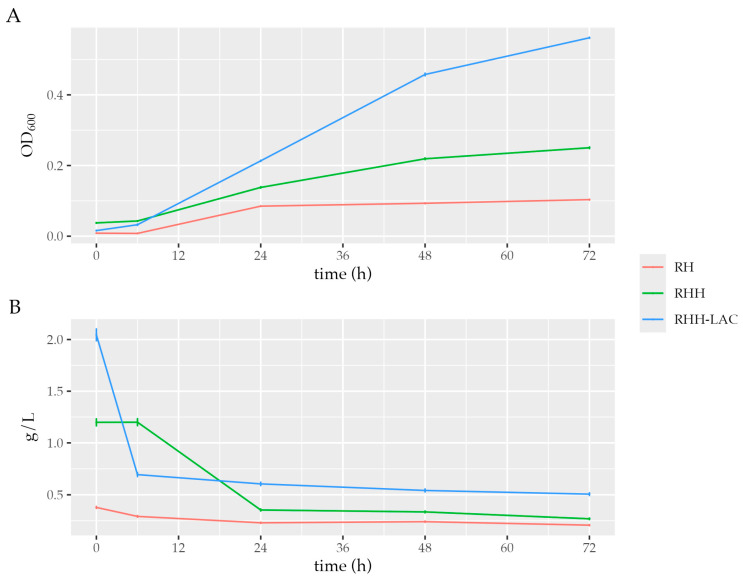
*B. subtilis* AC7 growth and sugar consumption in rice husk-based media with different enzymatic pretreatments in a bench-scale bioreactor. (**A**) Growth curves (OD_600_) and (**B**) reducing sugar concentrations (expressed as glucose equivalents, g/L) in untreated rice husk medium (RH, red line), rice husk subjected to starch hydrolysis (RHH, green line), and rice husk pretreated with both starch hydrolysis and laccase (RHH-LAC, blue line) during 72 h of fermentation.

Growth monitoring (Figure 8A) revealed that *B. subtilis* AC7 reached the highest optical density in RHH-LAC, maintaining exponential growth until 48 h. After this point, the increase in OD_600_ became progressively slower, indicating that the culture was entering the stationary phase between 48 and 72 h. A slight increase in OD_600_ was still detected up to 72 h (OD_600_ = 0.56), reflecting the gradual slowdown of cell growth. In contrast, growth in RHH reached a maximum OD_600_ of 0.25 and entered the stationary phase after 24–48 h, while in RH the growth was minimal, with OD_600_ stabilizing at 0.10 and the stationary phase reached as early as 24 h.

Sugar consumption profiles (Figure 8B) supported these observations. At inoculation (t = 0), reducing sugar concentrations were highest in RHH-LAC (2.05 g/L) compared to RHH (1.20 g/L) and RH (0.38 g/L). During *B. subtilis* AC7 growth in RHH-LAC, reducing sugars decreased rapidly to 0.7 g/L within the first 6 h and then gradually stabilized around 0.5 g/L up to 72 h, indicating a sustained availability of carbon sources during growth. In RHH, the reducing sugar profile showed a depletion between 6 h and 24 h, reaching about 0.35 g/L and remaining nearly constant thereafter. In RH, reducing sugars decreased slightly during the first hours of growth and then stabilized at a value around 0.21 g/L over the 72 h.

Biosurfactant production was evaluated by measuring the surface tension of cell-free supernatants after 72 h (Table 10). The strongest effect was observed in RHH-LAC, where surface tension decreased from 53.8 to 34.9 mN/m (−35%), followed by RHH with 35.65 mN/m (−29%), while RH showed only a moderate reduction (−18%), achieving 43.42 mN/m.

#### Surfactin Quantification in Bioreactor Extracts from Rice Husk Media

Surfactin production in *B. subtilis* AC7 cultures grown in rice husk-based media was further investigated. Crude solvent extraction confirmed the trend observed in surface tension, with RHH-LAC yielding the highest amount of extract (914 mg/L), followed by RHH (383 mg/L) and RH (180 mg/L). Surfactin quantification was carried out by LC–ESI–MS/MS. Calibration curves were constructed using purified surfactin from LB medium as the standard, showing good linearity in the range of 0.1–100 mg/L (R^2^ = >0.99).

As shown in Table 11, all three tested crude extracts (RH, RHH, and RHH-LAC) contained detectable amounts of surfactin homologues, with C15 being the most abundant in every case. The highest total surfactin concentration was observed in RHH-LAC (237.5 mg/L), followed by RHH (153.7 mg/L) and RH (118.9 mg/L). Among homologues, C15-surfactin accounted for 61.2% in RHH-LAC, 74.3% in RHH, and 73.2% in RH, confirming its dominance across substrates. Interestingly, the proportion of C14-surfactin was higher in RHH-LAC (30.4%) compared to RHH (15.4%) and RH (15.9%).

When normalized to the extraction yield, RHH-LAC supported the highest recovery of surfactin (914 mg/L crude extract, of which 237.5 mg/L was surfactin), followed by RHH (383 mg/L extract, 153.7 mg/L surfactin) and RH (180 mg/L extract, 118.9 mg/L surfactin).

## 4. Discussion

In the present work, rice husk was used as a renewable and sustainable substrate to produce lipopeptides (mainly surfactin) by the endophytic strain *B. subtilis* AC7. These lipopeptides are already known for their anti-adhesive properties against *Candida albicans* biofilm formation [36,40,49]. Based on the rationale that lignocellulosic agricultural residues represent cost-effective alternatives to conventional media, this study combined two complementary experimental approaches aimed at improving lipopeptide production from rice husk-based media both in native form and after enzymatic hydrolysis. In addition, a preliminary scale-up from shake-flask to bench-scale fermentation was performed.

An initial shake-flask screening and optimization phase identified the cultivation parameters most closely associated with surface tension reduction and biosurfactant pro-duction by *B. subtilis* AC7. Subsequently, enzymatic pretreatment strategies were investigated to enhance the release of bioavailable compounds from the lignocellulosic matrix, to support more efficient microbial growth and lipopeptide production, and to obtain clarified rice husk hydrolysates more suitable for controlled bench-scale fermentation processes.

First, optimization of rice husk-based substrate was carried out in shake-flask experiments using two factorial experimental designs, which were applied to identify the variables most significantly influencing lipopeptide production.

The selection of variables (e.g., nitrogen source, pH, and mineral ions) was based on the previous literature highlighting their importance in biosurfactant biosynthesis [50,51]. Surface tension reduction (ΔT) at 24 h and 48 h was used as an indirect measurement for biosurfactant accumulation, a method frequently employed in medium optimization studies [52,53]. In addition, although incubation time was not an explicit design variable, its inclusion as a covariate provided preliminary indications of the temporal dynamics of lipopeptide synthesis. In the first design, NaNO_3_ supplementation was associated with the greatest surface tension reduction, followed by incubation time and (NH_4_)_2_SO_4_, although these effects were not statistically significant. Nitrogen sources may positively influence surfactin production via nitrogen assimilation pathways and global metabolic control [54]. On the other hand, increasing the pH to 7.5 and supplementing with MgSO_4_ + MnSO_4_, yeast extract, or soybean oil correlated negatively with ΔT. Such negative associations may reflect metabolic imbalances or nutrient interactions that limit surfactin synthesis due to excessive mineral or organic supplementation that can stimulate cell growth rather than metabolite production.

In the second experimental design, replacing NaNO_3_ with NH_4_NO_3_ resulted in a stronger and statistically significant positive effect on ΔT (*p* = 0.005), underlining the critical role of nitrogen in surfactin biosynthesis. Time also maintained a significant positive effect (*p* = 0.048), suggesting that surfactin accumulation is higher in later growth phases. Interestingly, NaCl had a significant negative effect (*p* = 0.037) on surface tension reduction. On the contrary, recent work showed that, despite optimal bacterial growth occurring without NaCl, biosurfactant production remained effective at 5% NaCl as suggested by surface tension values of 29 ± 0.85 mN/m, comparable to those recorded at 0% NaCl [55].

Although clear statistical significance was observed only for a limited number of parameters, the experimental designs overall indicated that NH_4_NO_3_ or NaNO_3_, an incubation time of 48 h, and a starting pH at 6.5 were the major factors positively affecting surface tension reduction, and thus surfactin production, in rice husk medium.

Direct-infusion mass spectrometry analyses confirmed that *B. subtilis* AC7 predominantly produces surfactin, whereas fengycin was detected only at low levels [36]. Consequently, method development and subsequent quantitative analyses were focused on surfactin. Application of the optimized method to LB-derived samples revealed that surfactin produced by *B. subtilis* AC7 is a heterogeneous mixture dominated by C15-surfactin, followed by C14- and C13-surfactin.

At the production level, a different trend was observed in rice husk-based media compared to LB. In unsupplemented rice husk medium (Sample 1), despite the relatively high amount of crude extract recovered, surfactin production was extremely low, indicating that rice husk alone is not sufficient to sustain efficient lipopeptide biosynthesis. Nitrogen supplementation significantly enhanced surfactin production, with media containing a single nitrogen source (Samples 4 and 5) achieving the highest titers, surpassing more complex formulations (Samples 2 and 3). These results are consistent with the positive effects of NaNO_3_ and NH_4_NO_3_ identified in the factorial designs. In addition, changes in surfactin homologue distribution were observed compared to LB, where C15-surfactin remained dominant but with a relative decrease in favor of C14- and C13-surfactin.

The analysis of extraction yield and surfactin purity further highlights the influence of the culture medium on both production and downstream recovery. LB medium resulted in the highest surfactin purity (76.1%), despite a moderate extraction yield (382.5 mg/L), indicating a more selective recovery in a chemically defined system. In contrast, rice husk-based media showed higher extraction yields (262.0–1632.0 mg/L) but much lower surfactin purity (1.6–18.0%), suggesting a matrix effect associated with lignocellulosic substrates. The co-extraction of a non-target compound is, in fact, a common challenge in lignocellulosic substrate-based fermentations, making the downstream extraction and purification of biosurfactants particularly complex [56]. In our study, the high proportion of co-extracted non-target compounds might have reduced the relative surfactin content in the crude extracts.

The supplementation experiments with *B. subtilis* AC7 confirmed that the nitrogen source strongly influences growth, pH dynamics, and biosurfactant production. Among the tested conditions, NaNO_3_ and NH_4_NO_3_ emerged as the most effective supplements, promoting both biomass accumulation and the greatest reduction in surface tension, suggesting enhanced lipopeptide synthesis. The delay in maximal surface tension reduction observed with NH_4_NO_3_ suggests a slower but sustained biosurfactant production, consistent with its delayed growth dynamics. Previous findings highlight that nitrogen limitation and nitrogen metabolic pathways strongly regulate surfactin yield [54]. Moreover, recent studies have demonstrated a correlation between increased surfactin synthesis, and the upregulation of proteins involved in nitrate uptake and reduction pathways in response to the presence of sodium nitrate [57].

The strong alkalinization observed in media containing NaNO_3_ or NH_4_NO_3_ (pH > 8.5) over the course of incubation aligns with known metabolic mechanisms: nitrate reduction and ammonium assimilation consume protons, thereby raising the pH of the medium. On the contrary, supplementation with (NH_4_)_2_SO_4_ resulted in slower and more limited growth, which may be explained by the decrease in pH to 5.2 observed under these conditions. In addition, the low reduction in surface tension (39–40 mN/m) also supports the idea that this nitrogen source is unsuitable for lipopeptide production by *B. subtilis* AC7, which is in line with another optimization study that reported that inorganic nitrogen sources containing nitrate support a higher surfactin yield than ammonium sulfate [58]. Similarly, it has been reported that ammonium ions, when provided as the sole nitrogen source, are insufficient to sustain robust surfactin production, promoting mainly biomass formation, while nitrate is preferentially consumed during secondary metabolism, when secondary metabolite synthesis occurs [57,59].

The lignocellulosic nature of rice husk represents a significant obstacle to microbial growth and metabolite production due to the limited accessibility of carbohydrates and nutrients. In the context of biosurfactant production, pretreatment of lignocellulosic agro-industrial residues represents a key step for improving substrate valorization [18,20]. In this study, enzymatic pretreatment with recombinant laccase rPOXA1b enhanced the capacity of rice husk-derived media to support *B. subtilis* AC7 growth in a dose-dependent manner. A progressive increase in growth observed at 80 and 100 U/g of laccase suggests that sufficient enzyme loading is required to achieve effective modification of the rice husk matrix. In lignocellulosic biomasses, lignin acts as a protective barrier surrounding cellulose and hemicellulose, thereby limiting polysaccharide accessibility and making saccharification and microbial access to nutrients more difficult [60].

Mechanistically, rPOXA1b laccase acts on lignocellulosic substrates by promoting lignin oxidation and modification, thereby reducing biomass recalcitrance and increasing the accessibility of polysaccharide fractions to subsequent enzymatic hydrolysis [42]. Previous works showed that laccase pretreatment of agro-industrial matrices modifies or removes lignin polymers, thereby altering the structure of hemicellulose and cellulose, enhancing subsequent saccharification and fermentation steps [42,61,62]. The complementary action of α-amylase and glucoamylase further enhanced the hydrolysis of the starch-containing fraction of rice husk, releasing additional fermentable sugars that complement the monosaccharides made available by laccase-mediated delignification. This sequential enzymatic strategy provides a mild and eco-friendly approach that selectively converts starch into simpler, bioavailable forms without generating inhibitory or toxic by-products [20].

In this study, the effect of enzymatic pretreatment was evident from the quantitative comparison of sugar availability and surface activity in shake-flask experiments. At the beginning of fermentation, RHH-LAC contained the highest concentration of reducing sugars (2.6 g/L), corresponding to approximately a 6.5-fold increase compared with untreated RH (0.4 g/L) and a 1.7-fold increase compared with RHH (1.5 g/L). This level of sugar release is comparable to values reported for other combined pretreatment approaches applied to lignocellulosic agro-industrial residues; for example, Ref. [35] reported sugar concentrations of 2.16 g/L in pretreated rice husk liquid using a combined alkaline and hydrothermal method, while defatted rice bran under the same conditions yielded up to 7.08 g/L, reflecting the strong influence of substrate composition on pretreatment efficiency. The higher availability of fermentable substrates was also associated with a stronger reduction in surface tension, which decreased to approximately 40 mN/m within 24 h in RHH-LAC, whereas RH and RHH remained around 60 mN/m throughout the 72 h incubation, indicating limited biosurfactant production under these conditions.

In parallel, batch cultures grown in RHH-LAC for up to 72 h exhibited greater growth, as evidenced by higher final OD_600_ values, and a greater metabolic activity reflected by an increase in pH (>8 at 24 h) and the rapid consumption of reducing sugars during the first six hours of incubation. In contrast, untreated RH and RHH supported lower growth, indicating that neither the untreated substrate nor starch hydrolysis alone provided the same nutritional suitability as the combined laccase-mediated pretreatment and saccharification. This is consistent with previous studies reporting that the sequential pretreatment and hydrolysis process applied to lignocellulosic food wastes increases fermentable sugar availability, resulting in higher butanol and ethanol production yields [63,64].

Building on the shake-flask screening and enzymatic pretreatment experiments, the selected rice husk-based media (RH, RHH, and RHH-LAC) were further evaluated in a 1 L bench-scale bioreactor. This step aimed to assess the feasibility of lipopeptide production from enzymatically pretreated rice husk media under controlled fermentation conditions, including regulated pH, defined aeration, and continuous monitoring of dissolved oxygen (DO) dynamics.

In RHH-LAC, extended exponential growth, DO consumption, and gradual pH increase indicated sustained metabolic activity and efficient substrate utilization. The combined enzymatic pretreatment delayed the transition to the stationary phase compared to untreated or partially hydrolyzed substrates. In contrast, RH and RHH exhibited rapid DO saturation, suggesting carbon limitation and reduced tricarboxylic acid cycle activity. The DO oscillations observed in RHH-LAC between 10 and 40 h likely reflect metabolic adaptation to the sequential uptake of soluble carbohydrates released by the combined laccase and amylolytic pretreatment, consistent with the established influence of DO on lipopeptide synthesis in *Bacillus* spp. [65].

Furthermore, the observed alkalinisation in RHH-LAC suggests active amino acid deamination and protein turnover, creating a biochemical environment that favors secondary metabolic pathways; such non-ribosomal peptide synthetase-mediated processes are known to be strongly influenced by the bacterial metabolic state, with optimal yields observed at controlled growth rates and specific carbon source availability [50,66,67].

From a process engineering perspective, the differences in DO profiles suggest distinct oxygen transfer requirements for each medium. For instance, RH requires minimal aeration due to the low growth rate of *B. subtilis* AC7, whereas RHH-LAC demonstrates a higher oxygen transfer rate demand, suggesting that agitation and airflow may need optimization for further scale-up. Maintaining DO levels at 40–60% saturation is likely optimal to prevent oxygen limitation during the peak fermentation window (10–48 h), as supported by modelling studies [66,67].

The superior growth kinetics of RHH-LAC also suggest favorable applicability in fed-batch processes. Continuous or pulse-feeding of pre-hydrolyzed husk liquor could sustain metabolic activity beyond 72 h, potentially increasing cell density and extending the duration of lipopeptide biosynthesis. In addition, the stable sugar plateau observed after 6–24 h suggests metabolic steady-state potential, which is advantageous for implementing closed-loop control strategies for pH, DO, and substrate feed rate. Recent fermentation studies using agricultural product wastes as alternative carbon sources for *B. subtilis* suggest that fed-batch strategies can substantially support lipopeptide production [68].

The positive effect of enzymatic pretreatment on the production of surface-active metabolites under controlled conditions was supported by surface tension measurements. After 72 h of bioreactor fermentation, a progressive decrease in surface tension was observed, with reductions of 18% in RH, 29% in RHH, and 35% in RHH-LAC.

Finally, surfactin quantification confirmed the previous observations. The highest yield was obtained in RHH-LAC (237.5 mg/L), followed by RHH (153.7 mg/L) and RH (118.9 mg/L). Under shake-flask conditions, in the first set of experiments, the highest surfactin titer in untreated rice husk medium supplemented with NaNO_3_ was slightly higher (266.4 mg/L; Table 7, Sample 4). However, this comparison should be interpreted cautiously, as the experiments differed in scale, medium configuration, and fermentation control conditions. Nevertheless, the bioreactor results confirmed the suitability of the optimized rice husk-based media under controlled fermentation conditions. Furthermore, DO profiles suggested that oxygen transfer was not limiting under the selected operating conditions, supporting the feasibility of the scale-up strategy and providing a basis for further process optimization.

To the best of our knowledge, the combined application of a recombinant fungal laccase and amylolytic enzymes to rice husk to support surfactin production by *B. subtilis* has not been previously described. This approach represents a dual-target enzymatic strategy aimed at improving both the structural lignocellulosic fraction and the nutritional starch-containing fraction of rice husk within a single pretreatment workflow. However, several studies have reported the production of crude biosurfactants from agro-industrial and lignocellulosic residues. For example, Rane et al. [69] reported crude biosurfactant production by *B. subtilis* ANR 88 using agro-industrial substrates, with higher production observed with molasses (0.241 g/L) compared to sugarcane bagasse (0.127 g/L) and orange peel (0.089 g/L). After Plackett–Burman design optimization, crude biosurfactant production with molasses increased to 0.746 g/L. Comparable strategies based on lignocellulosic hydrolysates have also been described by Cortés-Camargo et al. [70], who used vine-trimming waste hydrolysates supplemented with mineral salt medium in a 2 L bioreactor, obtaining 1.52 g/L of crude extracellular biosurfactant from *B. tequilensis* ZSB10.

A relevant study by Khondee et al. [35] reported a crude biosurfactant concentration of 0.799 ± 0.064 g/L from rice husk using *Brevibacterium casei* NK8. In their process, rice husk was subjected to combined alkaline and hydrothermal pretreatment, and biosurfactant production was then carried out in a non-neutralized alkaline medium containing both the pretreated liquid and the solid residues.

Beyond the total surfactin yield, the bioreactor extracts were also compared in terms of surfactin homologue distribution. Surfactin C15 was the predominant isoform in all three extracts. Notably, the increased proportion of surfactin C14 in RHH-LAC suggests that greater nutrient availability affects the distribution of different isoforms. It is well documented that the type and quantity of surfactin homologues produced by *B. subtilis* are significantly influenced by growth conditions. For example, the use of alternative carbon sources, such as cellobiose, starch, and mannitol, instead of glucose has been shown to influence the proportion of different surfactin variants produced by *B. subtilis* SZMC 6179J [71]. Similarly, in fermentations using agro-industrial residues, such as crude glycerol or rapeseed and sunflower cakes, surfactin isoform proportions vary depending on feedstock composition [72,73].

Although the reduction in surface tension observed in the optimized rice husk media (~33–34 mN/m), together with LC-MS/MS analyses, clearly confirmed surfactin production by *B. subtilis* AC7, these values indicate a more moderate surface activity compared to some highly efficient surfactin-producing *Bacillus subtilis* strains cultivated under fully optimized conditions [9,73,74,75,76]. This result may be related to the chemical nature of the crude extracts and to the use of lignocellulosic agro-industrial substrates [77,78]. Specifically, rice husk is characterized by a complex composition rich in lignin, silica, and structural polysaccharides, which may limit nutrient accessibility and microbial metabolism, even after enzymatic pretreatment [79]. In addition, lignocellulose-derived compounds generated during thermal processing may partially inhibit microbial activity or biosurfactant biosynthesis, as previously reported for *Bacillus subtilis* cultivated in lignocellulosic hydrolysates [80].

Another important aspect may be represented by oxygen transfer and nutrient balance. Surfactin production is strongly influenced by aeration, dissolved oxygen availability, and carbon/nitrogen balance [81], which are parameters that were not fully optimized in the present study. Nevertheless, the observation that RHH-LAC supported higher biomass formation, prolonged metabolic activity, and improved surfactin production indicates that substrate accessibility and carbon availability play a key role in process efficiency, even under non-optimized cultural conditions.

To address these current limitations and fully exploit the process potential, future development strategies could include further refinement of enzymatic pretreatment conditions, optimization of C/N balance and trace element supplementation, fed-batch fermentation approaches, and advanced bioreactor aeration/agitation control to improve oxygen transfer and surfactin yields [66]. In addition, further studies should investigate the optimization of fermentation time in order to better correlate surfactin biosynthesis with the different growth phases of *B. subtilis* AC7 and identify the most productive harvesting point. Considering the intrinsic variability of agro-industrial residues, the evaluation of rice husk derived from different rice varieties and cultivation conditions may also help identify substrates with more favorable compositions for biosurfactant production and process sustainability [82]. Finally, optimization of downstream processing and extraction procedures, including the definition of selective purification workflows for surfactin recovery, could further improve product yield, purity, and process scalability, as downstream processing still represents one of the major economic bottlenecks in biosurfactant production [83,84]. Despite these limitations, the present results demonstrate the feasibility of valorizing rice husk as a renewable substrate for biosurfactant production within a sustainable circular bioeconomy framework [84].

## 5. Conclusions

This study demonstrates that rice husk is a promising, low-cost substrate for the sustainable production of surfactin. The Plackett–Burman experimental design for substrate optimization highlights the importance of nitrogen source, incubation time, and pH as crucial factors for *B. subtilis* AC7 surface tension reduction and, by inference, lipopeptide production.

The subsequent enzymatic treatment of rice husk, combining laccase with starch hydrolysis, further leveraged the substrate’s potential, supporting *B. subtilis* AC7 growth and surfactin production. Furthermore, the transition from flask to a bench-scale bioreactor suggested the reproducibility of the process under controlled operating parameters, confirming the potential applicability of this combined treatment for sustainable scale-up.

In conclusion, while further optimization is required to improve productivity, these findings provide a preliminary basis for developing circular, low-cost strategies that utilize lignocellulosic waste materials for value-added bioproducts.

## Figures and Tables

**Table 1:** Chemical analysis of rice husk used as substrate (Chamber of Commerce of Turin).

Parameter	Value	Method Reference
Moisture	12.73%	Reg. EC 152/2009 (27/01/2009 GU CE L 54 Annex III A)
Ashes	9.07%	Reg. EC 152/2009 (27/01/2009 GU CE L 54 Annex III M)
Ash Insoluble in HCl	0.43%	Dir. CEE 71/250 (15/06/1971 GU CEE L155/20 12/07/1971 pt. 6); Dir. CEE 81/680 (30/07/1981 GU CEE L246 29/08/1981 Annex pt. 10.1)
Proteins (N × 6.25)	14.37%	ISO 1871:2009 [37]
Sugars	6.8%	ISTISAN Reports 1996/34, pg. 63
Starch	19.43%	Reg. EC 900/2008 (16/09/2008 GU CE L248/7 17/09/2008 Annex I)
Crude Fat Substances	6.53%	MI0236 rev. 9/2009 Soxhlet extraction after hydrolysis
Fatty Acids—Saturated	1.12%	ISO 5508:1990 + EN ISO 5509:2000 [38,39]
Fatty Acids—Monounsaturated	2.89%	ISO 5508:1990 + EN ISO 5509:2000
Fatty Acids—Polyunsaturated	2.52%	ISO 5508:1990 + EN ISO 5509:2000
Neutral Detergent Fiber (NDF)	29.67%	M1150 rev. 2:2001
Acid Detergent Fiber (ADF)	10.56%	M1150 rev. 2:2001
Acid Detergent Lignin (ADL)	5.71%	M1150 rev. 2:2001

**Table 2:** First PB experimental design for the study of *B. subtilis* AC7 lipopeptide production comprising 12 experiments.

Variables	Lower Level (−1)	Higher Level (1)	Experiments											
			1	2	3	4	5	6	7	8	9	10	11	12
Yeast extract *	0	5	1	−1	−1	−1	1	−1	1	−1	1	−1	1	1
Soybean oil *	0	15	−1	1	−1	−1	1	1	1	−1	−1	1	−1	1
(NH_4_)_2_SO_4_ *	0	2.5	−1	−1	1	−1	−1	1	1	−1	1	1	1	−1
MgSO_4_+MnSO_4_ *	0	0.4 + 0.15	−1	1	−1	−1	1	−1	−1	1	1	1	1	−1
NaNO_3_ *	0	3	1	1	1	−1	1	1	−1	−1	−1	−1	1	−1
pH	6.5	7.5	−1	−1	1	−1	1	1	−1	1	1	−1	−1	1

* Concentrations in g/L.

**Table 3:** Second PB experimental design for the study of *B. subtilis* AC7 lipopeptide production comprising 12 experiments.

Variables	Lower Level (−1)	Higher Level (1)	Experiments											
			1	2	3	4	5	6	7	8	9	10	11	12
KH_2_PO_4_ *	0	4	1	−1	−1	−1	1	−1	1	−1	1	−1	1	1
MgSO_4_ *	0	1	−1	1	−1	−1	1	1	1	−1	−1	1	−1	1
NaCl *	0	5	−1	−1	1	−1	−1	1	1	−1	1	1	1	−1
FeSO_4_ *	0	0.1	−1	1	−1	−1	1	−1	−1	1	1	1	1	−1
Glucose *	0	10	1	1	1	−1	1	1	−1	−1	−1	−1	1	−1
NH_4_NO_3_ *	0	3	−1	−1	1	−1	1	1	−1	1	1	−1	−1	1
Urea *	0	0.2	1	1	−1	−1	−1	1	1	1	1	−1	−1	−1

* Concentrations in g/L.

**Table 4:** Change in surface tension (ΔT) with respect to the baseline in cell-free supernatants of *B. subtilis* AC7 cultures in PB experimental design 1. Coded level −1 indicates the lower level and coded level 1 indicates the higher level reported in the table; for all factors except pH, the lower level corresponds to the absence of the component.

Experiment	Variables						ΔT = T_t_ − T_0_	
	Yeast Extract	Soybean Oil	(NH_4_)_2_SO_4_	MgSO_4_ + MnSO_4_	NaNO_3_	pH	24 h	48 h
1	1	−1	−1	−1	1	−1	−6.08	−4.43
2	−1	1	−1	1	1	−1	−8.96	−7.19
3	−1	−1	1	−1	1	1	−5.58	−4.79
4	−1	−1	−1	−1	−1	−1	−6.20	−6.96
5	1	1	−1	1	1	1	2.38	1.89
6	−1	1	1	−1	1	1	−4.78	−4.27
7	1	1	1	−1	−1	−1	−1.29	−3.49
8	−1	−1	−1	1	−1	1	−0.67	0.44
9	1	−1	1	1	−1	1	−2.28	−3.67
10	−1	1	1	1	−1	−1	−1.20	−3.28
11	1	−1	1	1	1	−1	−7.7	−7.12
12	1	1	−1	−1	−1	1	−4.92	−7.49

**Table 5:** Change in surface tension (ΔT) with respect to the baseline in cell-free supernatants of B. subtilis AC7 cultures in PB experimental design 2. Coded level −1 indicates the lower level and coded level 1 indicates the higher level reported in the table; for all factors except pH, the lower level corresponds to the absence of the component.

Experiment	Variables							ΔT = T_t_ − T_0_	
	KH_2_PO_4_	MgSO_4_	NaCl	FeSO_4_	Glucose	NH_4_NO_3_	Urea	24 h	48 h
1	1	−1	−1	−1	1	−1	1	0.02	−1.00
2	−1	1	−1	1	1	−1	1	−4.39	−8.23
3	−1	−1	1	−1	1	1	−1	−5.24	−6.88
4	−1	−1	−1	−1	−1	−1	−1	−6.20	−6.96
5	1	1	−1	1	1	1	−1	−3.40	−6.32
6	−1	1	1	−1	1	1	1	−2.15	−6.13
7	1	1	1	−1	−1	−1	1	−0.14	−3.12
8	−1	−1	−1	1	−1	1	1	−4.85	−5.74
9	1	−1	1	1	−1	1	1	−5.85	−6.27
10	−1	1	1	1	−1	−1	−1	2.49	−2.74
11	1	−1	1	1	1	−1	−1	−0.87	−1.30
12	1	1	−1	−1	−1	1	−1	−8.35	−9.05

**Table 6:** Estimated coefficients and *p*-values for the two PB designs in terms of coded variables.

Plackett–Burman	Factor	Estimate	*p*-Value
Design 1	time	−0.128	0.833
	Yeast extract	0.385	0.530
	Soybean oil	0.518	0.400
	(NH_4_)_2_SO_4_	−0.053	0.931
	MgSO_4_ + MnSO_4_	0.955	0.131
	NaNO_3_	−0.651	0.293
	pH	1.257	0.052
Design 2	time	−1.033	0.048
	KH_2_PO_4_	0.473	0.340
	MgSO_4_	−0.015	0.975
	NaCl	1.095	0.037
	FeSO_4_	0.323	0.511
	Glucose	0.453	0.360
	NH_4_NO_3_	−1.574	0.005
	Urea (g/L)	0.290	0.555

**Table 7:** LC–MS/MS MRM parameters for surfactin homologue analysis and relative abundance in the purified fraction from LB-grown cultures.

	Rt min	[M+Na]^+^ *m*/*z*	MS/MS (nce% ^1^) *m*/*z*	Abundance %	Calibration Curve *R*^2^	LOD and LOQ (mg/L)
AC7BS						
C13-surfactin	16.85	1030	707,786 (36)	11.63	0.9947	0.02/0.06
C14-surfactin	17.81	1044	707,800 (36)	18.16	0.9968	0.02/0.08
C15-surfactin	18.62	1058	707,814 (38)	70.21	0.9954	0.06/0.19
Total				100		

^1^ nce%: normalized collision energy.

**Table 8:** Amounts of surfactin in crude extracts from different substrates.

	Sample LB	Sample 1	Sample 2	Sample 3	Sample 4	Sample 5
Extraction yield (mg/L)	382.5	262.0	531.0	722.0	1480.0	1632.0
Surfactin purity %	76.1	1.6	9.00	8.00	18.00	16.9
Total surfactin (mg/L)	290.7	4.19	47.79	57.76	266.4	275.8

**Table 9:** Percentages of surfactin congeners in AC7BS crude extracts obtained from different growth media.

Surfactin Homologues	Sample LB (%)	Sample 1 (%)	Sample 2 (%)	Sample 3 (%)	Sample 4 (%)	Sample 5 (%)
C13-surfactin	11.11	21.24	20.00	22.02	20.00	20.00
C14-surfactin	16.29	32.93	32.99	32.58	32.59	33.60
C15-surfactin	72.60	45.82	47.00	45.39	47.40	46.40

**Table 10:** Surface tension (TS) and relative reduction (%) of cell-free supernatants of *B. subtilis* AC7 cultures, measured before (0 h) and after 72 h of bench-scale bioreactor fermentation. Values are reported as mean ± standard deviation.

Sample	Surface Tension (mN/m)		Surface Tension Reduction (%)
	Non-Fermented	Fermented	
RH	53.21 ± 0.09	43.42 ± 0.37	18
RHH	50.44 ± 0.96	35.65 ± 0.57	29
RHH-LAC	53.78 ± 0.66	34.86 ± 0.33	35

**Table 11:** Percentage of surfactin homologues and total surfactin production (mg/L) extracted from cell-free supernatants of *B. subtilis* AC7 cultures grown in different rice husk media during bench-scale bioreactor fermentation.

Surfactin Homologues (%)	RH	RHH	RHH-LAC
C13-surfactin	10.93	10.30	8.47
C14-surfactin	15.86	15.39	30.37
C15-surfactin	73.21	74.31	61.16
Total surfactin (mg/L)	118.96	153.69	237.50

## Data Availability

The original contributions presented in this study are included in the article. Further inquiries can be directed to the corresponding author.

## Abbreviations

The following abbreviations are used in this manuscript:

AC7BS	AC7 BioSurfactant
AUC	Area Under the Curve
BS	BioSurfactant
CFU/mL	Colony-Forming Units per Milliliter
CMC	Critical Micelle Concentration
DNS	3,5-DiNitroSalicylic acid
DO	Dissolved Oxygen
ESI	ElectroSpray Ionization
ESI–MS	ElectroSpray Ionization–Mass Spectrometry
ESI–MS/MS	ElectroSpray Ionization–Tandem Mass Spectrometry
LB	Luria–Bertani
LC–ESI-MS/MS	Liquid Chromatography–ElectroSpray Ionization–Tandem Mass Spectrometry
LC-MS	Liquid Chromatography–Mass Spectrometry
LC-MS/MS	Liquid Chromatography–Tandem Mass Spectrometry
LOD	Limits of Detection
LOQ	Limits of Quantification
MRM	Multiple Reaction Monitoring
m_x_	Main Effect
NCE	Normalized Collision Energy
OD_600_	Optical Density at 600 nm
PB	Plackett–Burman
RH	Rice Husk Medium
RHH	Alpha-amylase and Glucoamylase Pre-treatments
RHH-LAC	Laccase, α-Amylase, and Glucoamylase Pre-treatments
TLC	Thin-Layer Chromatography
